# Is DOTS-Plus a Feasible and Cost-Effective Strategy?

**DOI:** 10.1371/journal.pmed.0030350

**Published:** 2006-09-12

**Authors:** Paul Garner, Marissa Alejandria, Mary Ann Lansang

## Abstract

Paul Garner and colleagues discuss the implications of the pilot study by Katherine Floyd and colleagues of the DOTS-Plus project for tuberculosis control in the Philippines.

Multidrug-resistant tuberculosis (MDR-TB) is on the increase, with several Eastern European countries having a prevalence of more than ten percent [1]. This resistance means that patients may not be cured with standard regimens even if they adhere well to treatment. An MDR-TB programme requires advanced medical care, infection control measures, counselling, and good follow-up, as one centre in the Philippines reports in a study published in *PLoS Medicine* [2].

The DOTS-Plus (directly observed treatment, short-course) project is run by the Makati Medical Center, supported by the Tropical Disease Foundation. The Center has exceptional management capacity, and was one of the first few nongovernmental organizations to be a principal recipient of grants from the Global Fund to Fight AIDS, Tuberculosis and Malaria (known as the Global Fund). The Center itself provides high-quality tertiary care. Within this setting, clinicians from the Makati Medical Center used drug history and sensitivity testing to provide individualised regimens of expensive second-line drugs, estimated to cost more than US$1,500 per patient. The authors report on a cohort of 171 patients with MDR-TB, of whom 117 were enrolled and analysed, with 61 percent cured. Indirect outcome data from patients from other countries were then combined with local cost data to estimate the number of premature deaths and secondary cases averted in order to provide an estimate of the mean cost per disability-adjusted life year gained. The 13 authors, from the Makati Medical Center, the Philippines Department of Health, the World Health Organization, and the Stanford University School of Medicine, conclude that treating MDR-TB patients is feasible and cost-effective in low- and middle-income countries.

The World Health Organization and other agencies are promoting services to treat MDR-TB [3]; the Global Fund is waiting in the wings, able to fund them. This promotion may cause pressure for countries to rapidly implement and expand such programmes. It is therefore imperative that expansion be based on sound evidence that such programmes work when scaled up. Hence, policy makers need to carefully appraise the evidence rather than just the conclusions of this paper and other papers, and consider the following four points.

First, this present analysis is not an independent evaluation. As is clearly stated, the authors include World Health Organization staff (who are promoting DOTS-Plus) and Tropical Disease Foundation staff (who are receiving Global Fund money for providing services). Further evaluations are needed to boost the evidence base.

Second, readers need to consider whether these specialist medical services can actually be provided at scale. This paper reports on a relatively small programme provided under ideal conditions; the operation was not intended to be under usual programmatic service provision. To establish such centres of excellence elsewhere in the country, infrastructure and staff training costs, not included in the cost-effectiveness analysis, would be considerable. In the event that decision makers do scale up MDR-TB services, health policy and systems research will be important to systematically identify health systems barriers and constraints, monitor progress, and draw out lessons from such programmes [4].

Third, policy makers need to take into account the potential adverse effects of such large investments in one aspect of TB control. Human resource capacity in the public health sector is already stretched: would such a programme divert political attention, resource allocation, specialist medical attention, and public health management capacity away from first-line treatment of tuberculosis—and, indeed, other functions of primary care? The Stop TB Partnership recommends that DOTS-Plus programmes should be instituted where there are effective DOTS programmes in place. DOTS-Plus programmes are distinguished from DOTS by the provision of additional second-line TB drugs, given as individualised treatment according to case by case susceptibility, or, when diagnostic facilities are fewer, as empirical treatment in people that have presumed MDR-TB. It is paradoxical that it is in areas where TB control is poorly implemented that the prevalence of MDR-TB is likely to rise. Ensuring adherence to primary treatment of tuberculosis is hard work and needs good health service strategy and management. Although these aspects may be viewed as rather mundane, they are central to promoting public health and avoiding the development of drug resistance in the first place.

Fourth, although the Global Fund can provide money for drugs for expensive special programmes, these programmes establish a highly expensive recurrent cost commitment that will go beyond the five-year term of the Global Fund grant. These cost commitments raise questions about the sustainability of these programmes. Past experience with secondary care in low- and middle-income countries indicates that these costs burgeon, and when money is short these medical costs may consume the lion's share of the total TB budget, at the expense of primary-care provision.

This paper reports on a well-run pilot study. It is a start, but should not be used as the basis for global scaling-up of MDR-TB programmes. It highlights the need for robust, prospective, and independent evaluation of any further investment in MDR-TB control, with careful attention to the potential negative impact on the overall health system, particularly primary TB control, in terms of investment of time, money, and political attention. At the end of the day, it is the responsibility of national policy makers to maintain control of their own health-care system, whatever external experts are pushing and whatever funding is on offer. These responsibilities mean having to balance primary care with the need for difficult and expensive treatment for a few but increasing number of patients.

## Abbreviations

DOTS: directly observed treatment, short-course
MDR-TB: multidrug-resistant tuberculosis
